# Cellulose

**Published:** 1889-04

**Authors:** 


### CELLULOSE.

Cellulose is a fibrous substance produced in France by a secret mechanical process, from cocoanut husk. When compressed, its specific gravity is far less than that of cork. It is the lightest solid known and therefore invaluable for life belts and life mattresses, while in large quantities its buoyancy will make a ship nonsinkable. It is used to fill compartments in a ship's side, and serves to prevent water entering the ship through a hole made by a projectile or rock, because the instant the water enters, the cellulose is expanded by it to an impenetrable tightness. It does not decay or emit any odor, and has been packed in ship's compartments without undergoing any change. If a mass of cellulose be penetrated by a projectile, it will not be ignited by the friction. If the neck of a bottle full of water be filled with cellulose, it will effectually cork the botttle. It is coming largely into use in the French navy.

